# MicroRNA-29b-1 impairs *in vitro* cell proliferation, self-renewal and chemoresistance of human osteosarcoma 3AB-OS cancer stem cells

**DOI:** 10.3892/ijo.2014.2618

**Published:** 2014-08-22

**Authors:** RICCARDO DI FIORE, ROSA DRAGO-FERRANTE, FRANCESCA PENTIMALLI, DOMENICO DI MARZO, IRIS MARIA FORTE, ANTONELLA D’ANNEO, DANIELA CARLISI, ANNA DE BLASIO, MICHELA GIULIANO, GIOVANNI TESORIERE, ANTONIO GIORDANO, RENZA VENTO

**Affiliations:** 1 Laboratory of Biochemistry, Department of Biological, Chemical and Pharmaceutical Sciences and Technologies, University of Palermo, Polyclinic, Palermo, Italy; 2 INT-CROM, ‘Pascale Foundation’, National Cancer Institute - Cancer Research Center, Mercogliano, Avellino, Italy; 3 Laboratory of Biochemistry, Department of Experimental Biomedicine and Clinical Neurosciences, University of Palermo, Polyclinic, Palermo, Italy; 4 Institute for Cancer Research and Molecular Medicine and Center of Biotechnology - College of Science and Biotechnology, Temple University, Philadelphia, PA, USA; 5 Department of Human Pathology and Oncology, University of Siena, Policlinico ‘Le Scotte’, Siena, Italy

**Keywords:** osteosarcoma, cancer stem cells, microRNA, microRNA-29b-1, multidrug resistance, 3AB-OS cells

## Abstract

Osteosarcoma (OS) is the most common type of bone cancer, with a peak incidence in the early childhood. Emerging evidence suggests that treatments targeting cancer stem cells (CSCs) within a tumor can halt cancer and improve patient survival. MicroRNAs (miRNAs) have been implicated in the maintenance of the CSC phenotype, thus, identification of CSC-related miRNAs would provide information for a better understanding of CSCs. Downregulation of miRNA-29 family members (miR-29a/b/c; miR-29s) was observed in human OS, however, little is known about the functions of miR-29s in human OS CSCs. Previously, during the characterization of 3AB-OS cells, a CSC line selected from human OS MG63 cells, we showed a potent downregulation of miR-29b. In this study, after stable transfection of 3AB-OS cells with miR-29b-1, we investigated the role of miR-29b-1 in regulating cell proliferation, sarcosphere-forming ability, clonogenic growth, chemosensitivity, migration and invasive ability of 3AB-OS cells, *in vitro*. We found that, miR-29b-1 overexpression consistently reduced both, 3AB-OS CSCs growth in two- and three-dimensional culture systems and their sarcosphere- and colony-forming ability. In addition, while miR-29b-1 overexpression sensitized 3AB-OS cells to chemotherapeutic drug-induced apoptosis, it did not influence their migratory and invasive capacities, thus suggesting a context-depending role of miR-29b-1. Using publicly available databases, we proceeded to identify potential miR-29b target genes, known to play a role in the above reported functions. Among these targets we analyzed CD133, N-Myc, CCND2, E2F1 and E2F2, Bcl-2 and IAP-2. We also analyzed the most important stemness markers as Oct3/4, Sox2 and Nanog. Real-time RT-PCR and western-blot analyses showed that miR-29b-1 negatively regulated the expression of these markers. Overall, the results show that miR-29b-1 suppresses stemness properties of 3AB-OS CSCs and suggest that developing miR-29b-1 as a novel therapeutic agent might offer benefits for OS treatment.

## Introduction

Osteosarcoma (OS), a highly aggressive tumor with a potent metastasizing potential, is the most common form of childhood cancer, comprising 2.4% of all malignancies in pediatric patients, and ~20% of all primary bone cancers (1,2). The current standard chemotherapy regimen (cisplatin, doxorubicin and methotrexate) provides only 65–70% long-term disease-free survival for OS patients without metastasis (3). Moreover, there is no established second-line chemotherapy for relapsed OS (4).

It is universally acknowledged that a successful cure of cancer requires the eradication of cancer stem cells (CSCs) (5), a subpopulation of cells which is the source for tissue renewal and hold malignant potential (6,7), and which confers resistance to therapies.

Previously (8), treating the human OS MG63 cells with 3-aminobenzamide (3AB), a potent inhibitor of poly(ADP-ribose) polymerase (PARP), we produced, isolated and patented for the first time a human OS CSC line which has been termed 3AB-OS. 3AB-OS cells are a heterogeneous and stable cell population which possesses properties (self-renewal and pluri-potency *in vitro*, tumorigenicity *in vivo*) that indicated them as CSCs (9,10). Moreover, they also express a large number of genes required for maintaining stemness, controlling cell cycle (in particular G1-S/G2-M phases progression) and inhibiting apoptosis. 3AB-OS CSCs have been characterized at genetic and molecular level (11). In comparison with parental MG63 cells, they are hypertriploid with a higher chromosome number ranging from 71 to 82. They also exhibit 49 copy number variations spanning almost all the chromosomes and 3,512 dysregulated genes. Moreover, they exhibit 189 differentially expressed (up-/downregulated) microRNAs (miRNAs).

MiRNAs are a novel class of small non-coding RNAs that regulate gene expression at the translational or post-transcriptional level by repressing translation from protein-encoding messenger RNAs (mRNAs) or by promoting degradation of their target mRNAs (12). Many studies have shown that miRNAs are aberrantly regulated in human cancers, suggesting a role as a novel class of oncogenes/tumor suppressor genes (13). MiRNA expression profiles can distinguish tumors from corresponding normal tissues and can suggest their developmental origin and differentiation state (14,15). Several studies have also shown that miRNAs are involved in the self-renewal and fate decisions of stem cells and that mechanism regulating the self-renewal nature of stem cells are dysfunctional in CSCs (16–20). Deregulation of miRNAs was recently reported in human OS (21–23) and it has been demonstrated that downregulation of miRNA-29 family members (miR-29a/b/c; miR-29s) is a frequent event evidenced in OS tissues (23). It has even been reported that the forced expression of miR-29s in OS cells inhibits cell proliferation and promotes cell apoptosis (24). However, little is known about the functions of miR-29s in human OS CSCs.

Our previous studies (11) have shown that, among the up-/downregulated miRNAs present in 3AB-OS cells, miR-29b-1 was highly downregulated. As targeting CSCs might permit a successful cure of OS, we believe that the knowledge of the role of miR-29b-1 in the regulation of cell growth, self-renewal and apoptosis in 3AB-OS CSCs might provide a new avenue for therapeutic interventions. Thus, in the present study, we examined the potential role of miR-29b-1 in 3AB-OS cells, by evaluating the *in vitro* effects of its functional overexpression.

## Materials and methods

### Cell culture

The human OS 3AB-OS CSCs were produced in our laboratory and patented (8,10). Cells were cultured as previously described (11).

### Vector construction for miR-29b-1 expression and stable transfection

A 498-bp insert from the *Homo sapiens* chromosome 7 genomic sequence (GenBank EU154353.1) containing the mir-29b-1 gene (MI0000105) were obtained through PCR from 100 ng of genomic DNA derived from the human HT29 colon cancer cell line. Amplification was performed with Pfu Ultra II fusion HS DNA polymerase (Stratagene, Agilent Technologies, Santa Clara, CA, USA) following the manufacturer’s instructions. The following primer pairs were used, in which we included *Eco*RI and *Not*I restriction sites for mir-29b-1: mir-29b-1-for: 5′-CGATAGCGAATTCGCTGAA CCTTTGTCTGGGC-3′; mir-29b-1-rev: 5′-TTCATTAGCGG CCGCGATCACAGTTGGATCCG-3′. The corresponding mir-29b-1 PCR fragments was digested with *Eco*RI/*Not*I and cloned into a plasmid, named pCDomH, derived from the pCDH-CMV-MCS-EF1-copGFP (System Biosciences, Mountain View, CA, USA) in which we inserted a fragment containing puromycin resistance that was obtained from the pmiRZip vector (System Biosciences) through a *Pst*I/*Kpn*I digestion. pCDomH plasmid, containing mir-29b-1, was sequence verified (BioRep S.r.l., Milan, Italy).

3AB-OS cells were plated in 6-well dishes until they reached 90% confluence and then transfected with pCDH-CMV-MCS-EF1-copGFP-T2A-PURO-miR-29b-1 or empty vector as a control (hereafter indicated as 3AB-OS-miR-29b-1-GFP cells and 3AB-OS-GFP cells, respectively), using Lipofectamine 2000 (Invitrogen, Life Technologies Ltd., Monza, Italy) according to the manufacturer’s instructions. Two days after transfections the cells were transferred into 100-mm dishes in selective medium containing 1 μg/ml puromycin (Santa Cruz Biotechnology, Santa Cruz, CA, USA); the medium was replaced every 3–4 days. A plate of untrasfected cells was used as a control for the selection. GFP (green fluorescent protein) expression of the transfected cells was assessed by fluorescence microscopy and flow cytometry to determine the transfection efficiency.

Fluorescence microscopy was performed using a Leica DM IRB fluorescence microscope (Leica Microsystems S.r.l., Milan, Italy) and images were photographed and captured by a computer-imaging system (Leica DC300F camera and Adobe Photoshop for image analysis. The GFP fluorescence was assayed employing a filter FITC set.

Flow cytometry analysis was performed by a Coulter Epics XL flow cytometer (Beckman Coulter S.r.l., Cassina De Pecchi, Milan, Italy) equipped with a single Argon ion laser (emission wavelength of 488 nm) and Expo 32 software. The green fluorescence was measured in the FL1 channel using a 515-nm BP filter.

### Growth curve and cell viability assays

Total cell number and viability were evaluated by trypan blue exclusion counting as previously described (25).

### Cell cycle and proliferation analyses

Cell cycle phase distribution was studied by flow cytometry of DNA content. For DNA staining, trypsinized cell suspensions were centrifuged, washed 3 times with PBS and resuspended at 1×10^6^ cells/ml in PBS. Cells were mixed with cold absolute ethanol and stored for 1 h at 4°C. After centrifugation, cells were rinsed 3 times in PBS and the pellet was suspended in 1 ml of propidium iodide (PI) staining solution (3.8 mM sodium citrate, 25 μg/ml PI, 10 μg/ml RNase A; Sigma-Aldrich S.r.l., Milan, Italy) and kept in the dark at 4°C for 3 h prior to flow cytometry analysis. The proliferation index was calculated as the sum of cells in S and G2/M phases of cell cycle (26). Flow cytometry analyses were performed by a Coulter Epics XL flow cytometer (Beckman Coulter) equipped with a single Argon ion laser (emission wavelength of 488 nm) and Expo 32 software. The red fluorescence was measured in the FL3 channel using a 620-nm BP filter. At least 1×10^4^ cells per sample were analyzed and data were stored in list mode files.

### Flow cytometry analysis of Ki-67 expression

For intracellular staining of Ki-67, at least 500,000 cells were processed using the Caltag Fix & Perm kit (Invitrogen) following the manufacturer’s guidelines. The antibodies used were FITC-conjugated anti-human/mouse Ki-67 and FITC-conjugated mouse IgG1k isotype control (BD Pharmingen, Buccinasco, Milan, Italy). Flow cytometry analysis was performed as reported above. The green fluorescence was measured as described in the above ‘Vector construction for miR-29b-1 expression and stable transfection’ paragraph. At least 1×10^4^ cells per sample were analyzed and data were stored in list mode files. Expression of cell marker was determined by comparison with isotype control.

### Three-dimensional (3D) cell culture

The 3D Culture BME (Cultrex, Trevigen; Tema Ricerca S.r.l., Bologna, Italy) was used in the assay. Briefly, BME gel was thawed on ice overnight at 4°C; 300 μl of 3D BME scaffold was seeded into 24-well plates and was then transferred to a CO_2_ incubator set at 37°C for 30 min to promote gel formation. Cells (2.0×10^4^) were seeded in DMEM (supplemented with 10% FBS) on top of the thick gel in each well.

Once plated on BME, all cultures were incubated at 37°C in a 5% CO_2_ humidified incubator for up to 14 days and media were replaced every 3 days. After 2 days, morphology was observed every 3 days via phase contrast microscopy using a Leica DM IRB inverted microscope (Leica Microsystems S.r.l.). Images were photographed and captured by a computer-imaging system (Leica DC300F camera and Adobe Photoshop for image analysis). Size of resulting structures were measured using ImageJ software.

### Sarcosphere and colony formation assay

These studies were performed as previously described (25).

### Chemosensitivity analysis

3AB-OS-miR-29b-1-GFP cells and 3AB-OS-GFP cells, were cultured to 150,000 cells/well in 6-well plates (Corning Costar, Euroclone, Pero, Italy) in culture medium. After 24 h cells were treated with 250 nM doxorubicin (Calbiochem, Millipore, Darmstadt, Germany), 10 μM cisplatin (Sigma-Aldrich) and 5 μM etoposide (Calbiochem, Millipore). Cell viability was analyzed by the trypan blue assay previously described (25). Apoptotic morphology was evaluated in cells stained with Hoechst 33342 (Sigma-Aldrich). In particular, cells were stained with Hoechst 33342 (2.5 μg/ml medium) for 30 min at 37°C and visualized by fluorescence microscopy using an appropriate filter for DAPI. Cells were evaluated on the basis of their nuclear morphology, noting the presence of homogeneous chromatin, condensed chromatin, and fragmented nuclei. Apoptosis was also studied by flow cytometry of DNA content as described in the above ‘Cell cycle and proliferation analyses’ paragraph. The proportion of cells giving fluorescence in the sub-G0/G1 phase of cell cycle was taken as a measure of apoptosis.

### Scratch/wound-healing and in vitro matrigel invasion assay

These studies were performed as previously described (25).

### RNA extraction and real-time RT-PCR

For miR-29b-1, total RNA extraction was performed using the Direct-zol RNA MiniPrep (Zymo Research, Euroclone); a DNase I treatment step was included. cDNA synthesis was carried out on 80 ng of total RNA, by using the mercury LNA™ Universal RT microRNA PCR kit (Exiqon, Euroclone), according to the manufacturer’s instructions. Afterwards, real-time PCR was performed, using 4 μl of cDNA product, miR-29b-1 LNA™ primers (204261; Exiqon), and SYBR Green master mix (Exiqon). PCR was performed under the following conditions: 95°C for 10 min, followed by 40 cycles of 95°C for 10 sec and 60°C for 1 min.

For Oct3/4, Sox2, Nanog, CD133, N-Myc, CCND2, E2F1, E2F2, Bcl-2 and IAP-2, 1 μg of total RNA was reverse transcribed by using the iScript™ cDNA Synthesis kit (Bio-Rad Laboratories S.r.l., Segrate, Milan, Italy), according to the manufacturer’s instructions. The resulting cDNAs were used for quantitative analysis by real-time PCR (qPCR) using the IQ SYBR Green Supermix (Bio-Rad) and the QuantiTect primers [QuantiTect Primer assay (200); Qiagen, Milan, Italy]. PCR primers used were: Oct3/4 (POU5F1: QT00210840), Sox2 (QT00237601), Nanog (QT01025850), CD133 (PROM1: QT00075586), N-Myc (QT00201404), CCND2 (QT00057575), E2F1 (QT00016163), E2F2 (QT00045654), BCL2 (QT00025011) and IAP-2 (BIRC3: QT00021798). PCR cycling was performed as follows: 95°C for 10 min; 95°C for 30 sec, 60°C for 60 sec, 72°C for 30 sec for 40 cycles and a final extension at 72°C for 5 min. All real-time PCR reactions are performed in triplicate. To ensure that the RNA samples were not contaminated with genomic DNA, we included a no reverse transcriptase control (no RT) during each run of real-time RT-PCR. Furthermore, to check the accuracy of amplifications, we included a negative control in each run by eliminating the cDNA sample in the tube. Real-time PCR and data collection were performed on an IQ5 cycler instrument (Bio-Rad) qPCR data were analyzed by IQ5 cycler software. The relative expressions of mRNAs and miRNAs were calculated using the comparative 2^−ΔΔCt^ method and were normalized using GAPDH (QT01192646; Qiagen) and U6 snRNA (203907; Exiqon), respectively.

### miRNA target prediction

Genes that contain the miR-29b-binding site(s) in the 3′-UTR were obtained using the TargetScan 5.1, MiRanda, PICTAR, miRbase and DIANA-microT target prediction algorithms, as previously described (11).

### Western blot analysis

Cells were washed in PBS and incubated on ice-cold lysis buffer (RIPA buffer 50 μl/10^6^ cells) containing protease inhibitor cocktail (Sigma-Aldrich) for 30 min and sonicated three times for 10 sec. Equivalent amounts of proteins (40 μg) were separated by SDS-polyacrylamide gel electrophoresis and transferred to a nitrocellulose membrane (Bio-Rad) for detection with primary antibodies against Oct3/4, Sox2, Nanog, N-Myc, CCND2, E2F1, E2F2, Bcl-2, IAP-2 (diluted 1:300; Santa Cruz Biotechnology), CD133 (diluted 1:250; Abgent, Flanders Court, San Diego, CA, USA) and the appropriate horseradish peroxidase-conjugated secondary antibodies. Immunoreactive signals were detected using enhanced chemiluminescence (ECL) reagents (Bio-Rad). The correct protein loading was confirmed by stripping the immunoblot and reprobing with primary antibody for actin (diluted 1:500; Sigma-Aldrich). Bands were visualized and photographed with Chemi Doc XRS (Bio-Rad). Quantification was performed using Quantity One software.

### Statistical analysis

Data, represented as mean ± SD, were analyzed using the two-tailed Student’s t-test using Microsoft Excel. Differences were considered significant at P<0.05.

## Results

### MiR-29b-1 overexpression reduces cell growth in 3AB-OS CSCs

**Figure 1:**
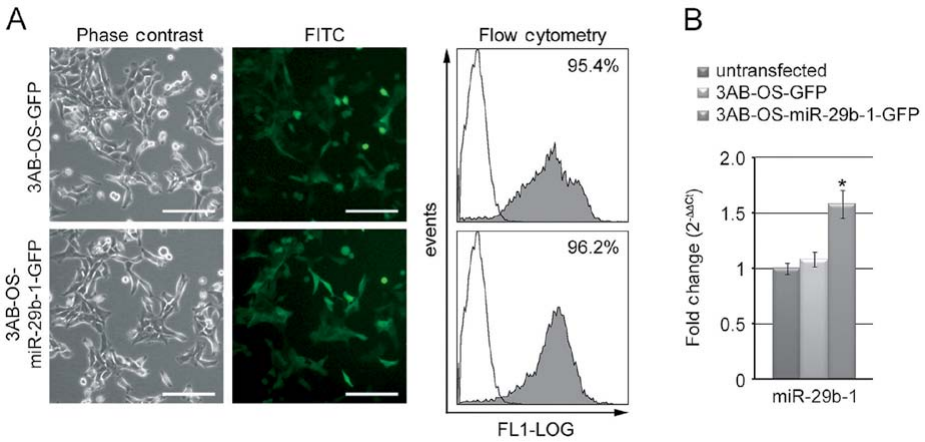
Evaluation of efficiency of miR-29b-1 transfection and expression in 3AB-OS cells. (A) Phase contrast (left panels) and fluorescence (middle panels) images of 3AB-OS-GFP cells (top panels) or 3AB-OS-miR-29b-1-GFP cells (bottom panels). The scale bar represents 100 μm. Cytometric analysis for the green fluorescent protein (GFP) expression (right panels) in 3AB-OS-GFP cells (top panel) and 3AB-OS-miR-29b-1-GFP cells (bottom panel). The open histogram indicates negative control (untransfected cells), filled histogram indicates the expression of GFP. Images are representative of four independent experiments. (B) Real-time RT-PCR analysis of miR-29b-1 in 3AB-OS untransfected cells, 3AB-OS-GFP cells and 3AB-OS-miR-29b-1-GFP cells. Data represent the mean with standard deviation (n=4); ^*^P<0.01 as compared to untransfected cells.

**Figure 2:**
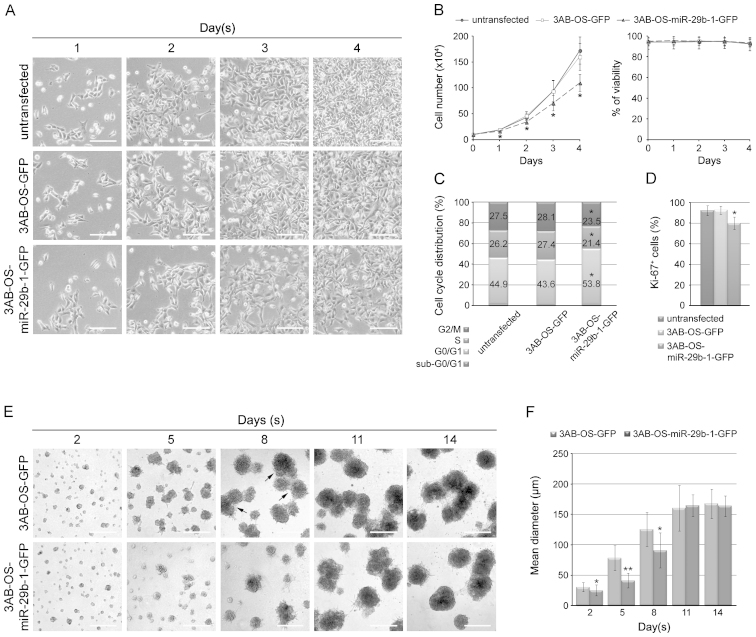
Effect of miR-29b-1 overexpression on growth and proliferation of 3AB-OS cells. (A) Phase contrast microscopy images at days 1–4 of untransfected cells (top panels), 3AB-OS-GFP cells (middle panels) and 3AB-OS-miR-29b-1-GFP cells (bottom panels) in culture. The scale bar represents 100 μm. Images are representative of four independent experiments. (B) Growth curves (cell number) and cell viability of untransfected cells, 3AB-OS-GFP cells and 3AB-OS-miR-29b-1-GFP cells. Data represent the mean with standard deviation (n=4); ^*^P<0.05 as compared to untransfected cells. (C) Cell cycle distributions determined using flow cytometry. Results are indicated as relative percentage of total cell cycle (^*^P<0.05, as untransfected cells). (D) Graph summarizing Ki-67 reactivity. Data represent the mean with standard deviation (n=4); ^*^P<0.05 as compared to untransfected cells. (E) Phase contrast microscopy images of 3AB-OS-GFP cells (top panels) and 3AB-OS-miR-29b-1-GFP cells (bottom panels) in a three-dimensional (3D) culture model on Matrigel. The black arrows indicate multi-cellular shere structures. The scale bar represents 200 μm. Images are representative of four independent experiments. (F) Graphs summarizing size of spherical masses from 3AB-OS-GFP and 3AB-OS-miR-29b-1-GFP cells cultured in 3D (on days 2, 5, 8, 11 and 14). Data represent the mean with standard deviation (n=4); ^*^P<0.05, ^**^P<0.01 as compared to 3AB-OS-GFP cells.

To examine the potential role of miR-29b-1 in 3AB-OS CSCs, as described in Materials and methods, we stably transfected 3AB-OS cells with either empty vector (3AB-OS-GFP cells) or vector containing miR-29b-1 (3AB-OS-miR-29b-1-GFP cells). To perform our study, preliminarily selected cells were used to evaluate the efficiency of miR-29b-1 transfection and expression. In comparison with phase contrast microscopy, fluorescence microscopy analysis (Fig. 1A) of the green fluorescent protein (GFP) shows a strong positivity for GFP homogeneously distributed in each group of transfected cells. Moreover, flow cytometry analysis confirmed a strong positivity for GFP (>95%). Real-time RT-PCR analysis in both 3AB-OS-miR-29b-1-GFP and 3AB-OS-GFP cells, in comparison with untransfected cells, shows increase in the expression of miR-29b-1 up to 1.55-fold (P<0.01) in 3AB-OS-miR-29b-1-GFP cells, while no significant variations were measured in 3AB-OS-GFP cells (Fig. 1B). Thereafter, we assessed the effect of miR-29b-1 overexpression on 3AB-OS cell proliferation. In Fig. 2A, phase contrast microscopy shows that cell number markedly decreased in 3AB-OS-miR-29b-1-GFP cells with respect to 3AB-OS-GFP and untransfected cells. In Fig. 2B cell count shows that miR-29b-1 overexpression markedly reduced the growth rate, whereas it did not induce loss of cell viability as shown by trypan blue exclusion assay. In agreement, studies of DNA content profiles, by flow cytometry analysis of propidium iodide stained cells, show that 3AB-OS-miR-29b-1-GFP cells were mostly in the G0/G1 phase, while untransfected and 3AB-OS-GFP cells were predominantly in S-G2/M (Fig. 2C). Moreover, analysis of the proliferation marker Ki-67 shows that 3AB-OS-miR-29b-1-GFP cells resulted to be less Ki-67-positive than untransfected and 3AB-OS-GFP cells (Fig. 2D). During our studies statistically significant difference between untransfected 3AB-OS cells and 3AB-OS-GFP cells were never observed (P>0.05). Therefore, we decided to employ 3AB-OS-GFP cells as control.

We analyzed the effects of miR-29b-1 overexpression in a three-dimensional (3D) culture model on Matrigel. As shown in Fig. 2E, 3AB-OS-miR-29b-1-GFP cells grew slower than 3AB-OS-GFP cells. Indeed, after 2 and 5 days in culture 3AB-OS-miR-29b-1-GFP cells formed spherical masses of cells smaller than that of 3AB-OS-GFP cells, suggesting a decrease of cell proliferation. After eight days, cell cluster density continued to increase in size, often appearing darker and denser; however, 3AB-OS-miR-29b-1-GFP clusters were much smaller than 3AB-OS-GFP clusters. Moreover, at this time, 3AB-OS-GFP clusters even generated multi-cellular sphere structures not evidenced in 3AB-OS-miR-29b-1-GFP clusters. From day 11 to 14, the structures of both cell lines gradually lost their spatial separation, tending to fuse into a single structure. Moreover, during this time they did not appreciably change in size, suggesting a cessation of proliferation. We even performed the progressive quantification of the sizes of the structures formed by the two cell lines in 3D. As shown in Fig. 2F, from day 2 to 8 the size of the structures resulting from 3D culture in Matrigel, were significantly different among the two cells lines. Indeed, at days 2, 5 and 8 the mean diameter of 3AB-OS-miR-29b-1-GFP structures (25.3±9, 41.3±12 and 90.9±28.6 μm, respectively) were smaller than those of 3AB-OS-GFP cells measured at the same times (30.5±7.5, 78.5±20.5 and 125.5±28 μm, respectively). At days 11 and 14, when the cell structures were stabilized and proliferation ceased, there was not significant difference among the two cells lines. At this stage, cell density might have reached the highest level, thus, the oxygen and nutrient supply by passive diffusion might have no longer been able to meet the need of the cell growth, nor to support the cell clusters to grow any more. Overall, the results suggest that in 3D culture 3AB-OS-miR-29b-1-GFP cells grow more slowly than 3AB-OS-GFP cells.

### MiR-29b-1 overexpression decreases self-renewal in 3AB-OS CSCs

**Figure 3:**
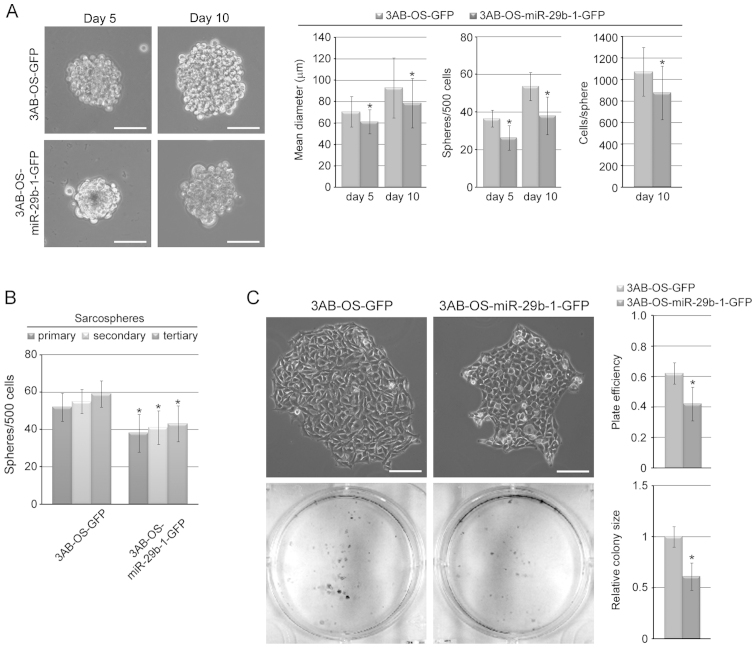
Effect of miR-29b-1 overexpression on sarcosphere- and colony-forming ability of 3AB-OS cells. (A) Phase contrast images of primary sarcospheres formed from 3AB-OS-GFP and 3AB-OS-miR-29b-1-GFP cells after 5 and 10 days of culturing. The scale bar represents 50 μm. Graphs summarizing size and number of sarcospheres from 500 cells (days 5 and 10) and number of cells/sphere on day 10. Data represent the mean with standard deviation (n=4); ^*^P<0.05 as compared to 3AB-OS-GFP cells. (B) Graph summarizing numbers of primary, secondary (generated from dissociated primary spheres) and tertiary (generated from dissociated secondary spheres) sarcospheres on day 10 from 500 cells. Data represent the mean with standard deviation (n=4); ^*^P<0.05 as compared to 3AB-OS-GFP cells. (C) Clonogenic growth of 3AB-OS-GFP and 3AB-OS-miR-29b-1-GFP cells after 10 days of culture. Phase contrast images (top; the scale bar represents 100 μm) and an image (bottom) of 6-well plate after staining with methylene blue. Graphs summarizing plate efficiency (colonies/100 cells) and relative colony size (mean area relative to 3AB-OS-GFP cells). Data represent the mean with standard deviation (n=4); ^*^P<0.05 as compared to 3AB-OS-GFP cells.

To test whether miR-29b-1 is important for 3AB-OS cells self-renewal, we tested, under non-adherent conditions (27), sarcosphere-forming ability of 3AB-OS-miR-29b-1-GFP cells compared to 3AB-OS-GFP cells. Fig. 3A shows that both cell lines were capable of forming sarcospheres. In particular, after days 5 in culture, 3AB-OS-GFP cells formed sarcospheres having a mean diameter of 70.5±14.2 μm, at a frequency of ~1/14 (36.6±4.5 spheres/500 cells), while 3AB-OS-miR-29b-1-GFP cells formed smaller sarcospheres (mean diameter of 61.2±11.3 μm) at a frequency of ~1/19 (26.3±6.5 spheres/500 cells). After 10 days, 3AB-OS-GFP sarcospheres increased in size and number, reaching a mean diameter of 92.9±28 μm, containing ~1,072 cells/sphere. Even 3AB-OS-miR-29b-1-GFP sarcospheres increased in size and number, but they were fewer in number and much smaller (mean diameter of 78.6±23 μm, containing ~875 cells/sphere). On analyzing sarcosphere-forming ability through subsequent passages (secondary and tertiary spheres), we found (Fig. 3B) that the number of sarcospheres generated from both cell lines in each passage remained consistent; however, 3AB-OS-miR-29b-1-GFP cells formed ~1.4-fold less sarcospheres than 3AB-OS-GFP cells, demonstrating that miR-29b-1 decreases the self-renewal capacity of sarcosphere-forming cells. In addition, in a colony-forming assay that correlates with self-renewal (28), 3AB-OS-miR-29b-1-GFP cells formed less numerous and smaller colonies than 3AB-OS-GFP cells (Fig. 3C). These data suggest that miR-29b-1 controls the growth and self-renewal capacity of 3AB-OS CSCs.

### MiR-29b-1 overexpression enhances the chemosensitivity of 3AB-OS CSCs

**Figure 4:**
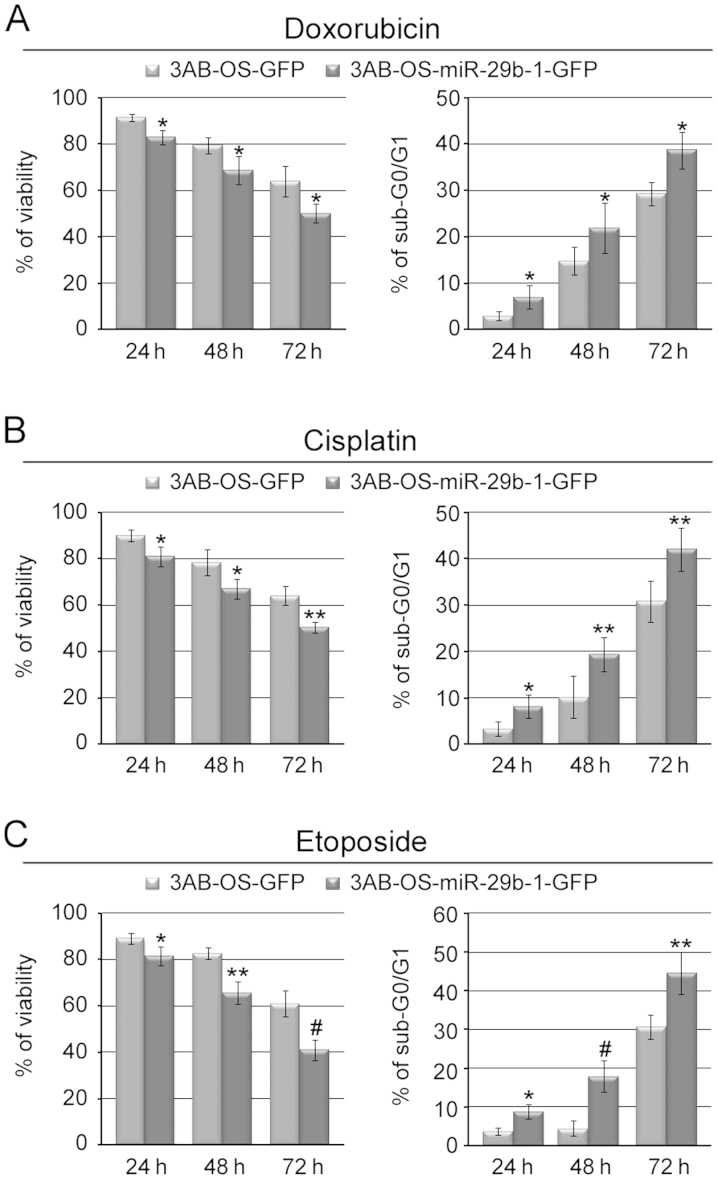
Effect of miR-29b-1 overexpression on chemosensitivity of 3AB-OS cells. Both 3AB-OS-GFP cells and 3AB-OS-miR-29b-1-GFP cells were treated with (A) doxorubicin (250 nM); (B) cisplatin (10 μM); and (C) etoposide (5 μM) for indicated times. Cell viability (left panels) was determined by trypan blue exclusion assay; apoptosis (right panels) was evaluated by flow cytometric analysis of propidium iodide DNA staining (percentages of cells in sub-G0/G1 phase). Data represent the mean with standard deviation (n=4); ^*^P<0.05, ^**^P<0.01 and ^#^P<0.005 as compared to 3AB-OS-GFP cells.

We next investigated whether miR-29b-1 could also enhance chemosensitivity of 3AB-OS cells. Fig. 4A and B (left panels) show that exposure of the cells to doxorubicin or cisplatin, two of the major drugs used for the chemotherapy of osteosarcoma (3,4), resulted in significant time-dependent reduced viability of 3AB-OS-miR-29b-1-GFP cells with respect to 3AB-OS-GFP cells. Furthermore, both morphological examination (data not shown) and flow cytometry assay of DNA content (percentage of cells in the sub-G0/G1 phase of cell cycle, taken as a measure of apoptosis) demonstrated that drug treatment induced in 3AB-OS-miR-29b-1-GFP cells a percentage of apoptosis much higher than in 3AB-OS-GFP cells (Fig. 4A and B, right panels). Fig. 4C shows that 3AB-OS-miR-29b-1-GFP cells were also much more sensitive to etoposide-induced apoptosis than 3AB-OS-GFP cells. These results suggest that miR-29b-1 may increase the sensitivity of 3AB-OS cells to different chemotherapeutic agents.

### MiR-29b-1 overexpression does not influence migratory and invasive capacities of 3AB-OS CSCs

**Figure 5:**
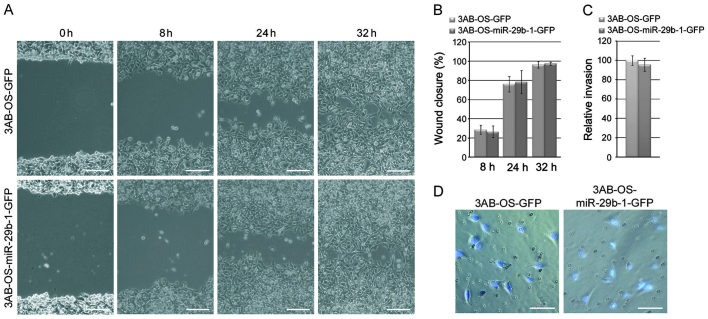
Effects of miR-29b-1 overexpression on migratory and invasion ability of 3AB-OS cells. (A) Representative images from the scratch wound-healing assay in 3AB-OS-GFP cells (top panels) and 3AB-OS-miR-29b-1-GFP cells (bottom panels). Cells were scratched and wound margins were imaged 0, 8, 24 and 32 h later. The scale bar represents 100 μm. (B) Quantification of the scratch wound-healing assay. The extent of wound closure was quantified by measuring the wound area compared to the initial wound area. Data represent the mean with standard deviation (n=4; P>0.05 as compared to 3AB-OS-GFP cells). (C) Graphs summarizing relative invasion (mean of the percentage of the number of cells relative to 3AB-OS-GFP cells). Data represent the mean with standard deviation (n=4; P>0.05 as compared to 3AB-OS-GFP cells). (D) Representative images from the transwell invasion assays in 3AB-OS-GFP and 3AB-OS-miR-29b-1-GFP cells. After 48 h of incubation, cells migrated to the underside of the insert were stained with Hoechst 33342. The scale bar represents 50 μm.

To evaluate whether miR-29b-1 overexpression influences the motility and invasivity of 3AB-OS cells, we performed scratch/wound healing and Matrigel Transwell invasion assays, respectively. In Fig. 5A and B the data from the wound-healing repair assay at 8, 24 and 32 h after scratching, show no significant differences (P>0.05) in migratory capacity between 3AB-OS-miR-29b-1-GFP cells and 3AB-OS-GFP cells. Similarly, no differences were observed in the cell invasive capacity between the two cell lines, as shown by Matrigel Transwell invasion assays (Fig. 5C and D).

### MiR-29b-1 overexpression reduces the expression of stemcell, cell cycle and anti-apoptotic markers in 3AB-OS CSCs

**Figure 6:**
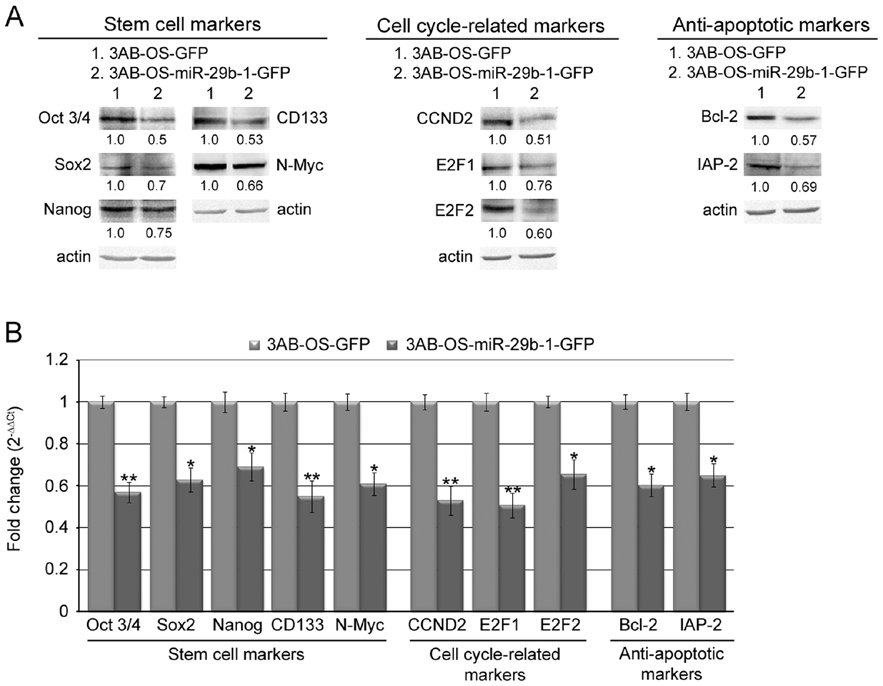
Effects of miR-29b-1 overexpression on stem-cell, cell cycle and anti-apoptotic marker expression in 3AB-OS cells. (A) Western blot analysis of stemness, cell cycle and anti-apoptotic proteins. Expression level relative to 3AB-OS-GFP cells is shown below the blots. The results are representative of four independent experiments. (B) Real-time RT-PCR analysis of mRNAs related to the above reported proteins. Data represent the mean with standard deviation (n=4); ^*^P<0.05 and ^**^P<0.01 as compared to 3AB-OS-GFP cells.

To predict the possible molecular target of miR-29b, we employed a number of avaible databases (TargetScan 5.1, MiRanda, PICTAR, miRbase and DIANA-microT). The analysis predicted a great number of targets know to be strong regulators of stemness, cell cycle and apoptosis (not shown). Among these we analyzed CD133, N-Myc, CCND2, E2F1 and E2F2, Bcl-2 and IAP-2, since they are overexpressed in 3AB-OS cells (8,11) and many of them were found to be frequently overexpressed in tissues of osteosarcoma patients (29–35). We also analyzed Oct3/4, Sox2 and Nanog, as they are the most important stemness markers previously found to be overexpressed in 3AB-OS CSCs (8,10). In Fig. 6A western blot analysis shows that in 3AB-OS-miR-29b-1-GFP cells protein levels of important stem cell markers (Oct3/4, Sox2, Nanog, CD133, N-Myc), cell cycle-related markers (CCND2, E2F1, E2F2) and anti-apoptotic markers (Bcl-2 and IAP-2) were markedly lower than in 3AB-OS-GFP cells. Moreover, real-time RT-PCR analysis (Fig. 6B) shows that, similarly, the level of mRNAs related to the above reported proteins were markedly lower in 3AB-OS-miR-29b-1-GFP cells than in 3AB-OS-GFP cells. These data suggest that miR-29b-1 may negatively regulate the expression of these markers and that its overexpression probably affects cell proliferation, self-renewal and chemosensitivity of 3AB-OS CSCs by directly or indirectly targeting their mRNAs.

## Discussion

MicroRNAs (miRNAs) are a class of non-coding regulatory RNAs of ~22 nucleotides (12) that are able to bind to specific sites typically present in the 3′-UTR of their target genes. They mediate either mRNA decay with perfect base pairing or translational blockade with imperfect base pairing (36). As miRNAs may act as oncogenes or tumor suppressor genes (13), they constitute a large gene regulatory network that can modulate proliferation, cancer, and stemness. This suggests that they might be novel biomarkers or therapeutic targets in cancer treatment. In recent years, it has been found that miRNAs are involved in tumorigenesis and carcer progression and that family of miR29s is aberrantly expressed in multiple cancers (37). A large body of studies has provided results on functions of miR29s in cancer, even suggesting their targeting for cancer therapy. Nevertheless, their functional mechanisms relevant to cancer are poorly understood.

It has been demonstrated that downregulation of the family of miR29s is a frequent event in OS tissues (23) and that its forced expression in OS cells inhibits cell proliferation and promotes cell apoptosis (24). However, their biological functions and possible mechanisms of action in OS CSCs have not been elucidated.

We have previously shown (11) that, in comparison with parental MG63 cells, 3AB-OS cells revealed miR-29b markedly downregulated. Here, we investigated the potential contribution of miR-29b-1 to 3AB-OS stemness. To perform these studies we upregulated miR-29b-1 in 3AB-OS cells; then, we examined the effects of this overexpression on cell proliferation, sarcosphere-forming ability, clonogenic growth, chemosensitivity, migration and invasive ability of 3AB-OS-miR-29b-1-GFP cells.

Our results demonstrated that in 3AB-OS-miR-29b-1-GFP cells proliferation was markedly reduced in both two- and three-dimensional culture systems. Furthermore, miR-29b-1 overexpression significantly downregulated protein and mRNA levels of its putative targets CCND2, E2F1 and E2F2. These putative targets are known to be involved in cell cycle regulation and DNA synthesis (38). Interestingly, it has been reported that miR-29s target CCND2 in various cancer types (39,40) and E2F1 in OS (24). E2F1 and E2F2 are members of the E2F family of transcription factors, and have been well-characterized as regulators of the G1-S phase transition (41). Previous reports indicate that E2F2 has strong oncogenic capacity and that cell lines transfected with E2F2 proliferate at twice the rate of control cells (42). For proper progression through cell cycle, phosphorylation activity of cyclin dependent kinase (Cdk) is essential. It is well known that CCNDs bind Cdk4 and Cdk6 (43) with consequent activation of Rb phosphorylation which inhibits Rb activity and activates E2Fs, allowing S-phase entry. Accordingly, overexpression of CCND2, E2F1 and E2F2 were reported in various cancer types, including OS (32,33).

In our previous study (8) we have shown that 3AB-OS cells have highly deregulated Rb function. Indeed, the analysis of its functional status evidenced that, in respect to parental MG63 cells, 3AB-OS cells express much higher levels of the hyperphosphorylated/inactive Rb form. Moreover, this is accompanied by CCND2 overexpression (11) and by very high levels of nuclear β-catenin (8) which also strongly correlated to cancer invasivity (44,45). Thus, the potent downregulation of miR-29b-1 in 3AB-OS cells might be at the root of their altered G1-S transition.

In this study, we also found that miR-29b-1 overexpression, in 3AB-OS CSCs, consistently reduced their sarcosphere-forming ability and colony formation. Moreover, in comparison with 3AB-OS-GFP cells, 3AB-OS-miR-29b-1-GFP cells also showed potently decreased stemness marker levels (Oct3/4, Sox2, Nanog, CD133 and N-Myc). Intriguingly, among them, CD133 and N-Myc are putative targets of miR-29b and CD133 is a recognized stem cell marker used for the identification and isolation of putative cancer stem cell populations from various malignant tumors, including OS (29,30). In particular, it is known that N-Myc gene has an essential role in normal hematopoietic stem cell function, and that in medulloblastoma genesis it is also responsible for the transformation of stem cells to CSCs (46,47). Oct3/4, Nanog, and Sox-2 are essential transcription factors critically involved in both self-renewal and maintenance of pluri/multipotency of undifferentiated embryonic/adult stem cells (48,49). Of great interest is that all of these genes are overexpressed in 3AB-OS cells and many of them were found to be frequently overexpressed in tissues of OS patients (31–33) and in stem cells isolated from OS cell populations (29,30). This suggests that expression of these genes may be a main feature of CSCs. Overall, these findings suggested that the deep downregulation of miR-29b-1 found in 3AB-OS CSCs might play a key role in regulating their stemness.

It is known that the reluctance of the cells to enter apoptosis could be an important cause of therapeutic resistance. We have previously shown that, in comparison with parental MG63 cells, 3AB-OS cells highly express a greater number of genes required for inhibiting apoptosis (FlipL, Bcl-2, XIAP, IAP1, IAP-2, and survivin) (8). Herein we show that miR-29b-1 overexpression sensitized 3AB-OS cells to chemotherapeutic drug-induced apoptosis and concomitantly decreased the expression of the anti-apoptotic genes Bcl-2 and IAP-2. The overexpression of Bcl-2 and IAP-2 has been identified in a variety of human cancers (50,51) and it has been reported that miR-29s target Bcl-2 in both hepatocellular carcinoma (HCC) and OS cell line (52,24).

Moreover, it has been shown (53) that miR-29b acts as an antimetastatic miRNA for prostate cancer cells at multiple steps in a metastatic cascade. However, in contrast, it has been shown that miR-29a can lead to epithelial-mesenchymal transition and metastasis in cooperation with oncogenic Ras signaling (54). This suggested that the role of miR-29s in cancer may depend on the context. Herein, our results showing that miR-29b-1 overexpression did not influence migratory and invasive capacities of 3AB-OS cells, agree with the role of the context in determining the effects of the family of miR-29s.

In conclusion, our study demonstrated that miR-29b-1 overexpression causes 3AB-OS CSCs proliferation, self-renewal and chemosensitivity. This is accompanied by downregulation of key stem cell markers (Oct3/4, Sox2, Nanog, CD133, N-Myc), cell cycle-related markers (CCND2, E2F1, E2F2) and anti-apoptotic markers (Bcl-2 and IAP-2). Overall, the results show that miR-29b-1 suppresses stemness properties of 3AB-OS CSCs and suggest that developing miR-29b-1 as a novel therapeutic agent might offer benefits for OS treatment.
